# War on fear: Solly Zuckerman and civilian nerve in the Second World War

**DOI:** 10.1177/0952695112470350

**Published:** 2012-12

**Authors:** Ian Burney

**Affiliations:** University of Manchester, UK

**Keywords:** blast, civilian neurosis, home front, inter-war psychology, Second World War, Solly Zuckerman

## Abstract

This article examines the processes through which civilian fear was turned into a practicable investigative object in the inter-war period and the opening stages of the Second World War, and how it was invested with significance at the level of science and of public policy. Its focus is on a single historical actor, Solly Zuckerman, and on his early war work for the Ministry of Home Security-funded Extra Mural Unit based in Oxford’s Department of Anatomy (OEMU). It examines the process by which Zuckerman forged a working relationship with fear in the 1930s, and how he translated this work to questions of home front anxiety in his role as an operational research officer. In doing so it demonstrates the persistent *work* applied to the problem: by highlighting it as an ongoing research project, and suggesting links between seemingly disparate research objects (e.g. the phenomenon of ‘blast’ exposure as physical and physiological trauma), the article aims to show how civilian ‘nerve’ emerged from within a highly specific analytical and operational matrix which itself had complex foundations.

In a 1934 review of Sir James Frazer’s *Fear of the Dead*, the anthropologist A. M. Hocart identified what for him was the key question that future analysts of early 20th-century Britain would have to answer: why was it ‘so fascinated by fear, why [was] that emotion made to account for everything?’ (cited in Kuklick, 1992: 119). Hocart was not wrong: fear loomed large in the writing of contemporary anthropologists, psychiatrists, sociologists and novelists, a fact not lost on subsequent generations of historians of inter-war Britain, who have tended to reach for phrases like ‘the age of anxiety’, ‘the age of insecurity’ and, most recently, the ‘morbid age’ to characterize the period (Overy, 2009). Equally cemented in national historical narrative, however, is the civilian wartime experience, in which the fearful inhabitants of the inter-war years transmuted into embodiments of British ‘nerve’. 

Up to a point, this is a story with familiar, intertwined elements: the legacy of shell shock and its attendant concerns about the management of fragile emotional economies (Shephard, 2000; Jones and Wessely, 2005); the emergence of an inter-war ‘culture of safety’ and its political ramifications (Overy, 2009); political, scientific and military accounts of a new era of ‘total war’, underscored by war reportage from Abyssinia, Manchuria and Spain, which in turn fed an apocalyptic popular culture fuelling notions of civilian vulnerability to air raids and emphasizing the catastrophic potential of mass panic (Patterson, 2007); and the rise of a popular psychology stressing over-civilized nervous subjects and the need for individual self-mastery (Thomson, 2006). 

This multi-layered discourse of fear had as one of its prime referents, of course, the prospect of a second catastrophic world war, a war in which psychic casualties were widely expected to outnumber physical ones. As the London-based psychoanalyst Edward Glover stated in a BBC radio broadcast:

… the whole atmosphere of modern war is likely to revive those unreasoning fears that the human race has inherited from its remotest ancestors; gas masks that make us look like strange animals; underground shelters; … enemies overhead and unseen; wailing sirens; screaming air bombs. … Small wonder, then, that we are afraid lest in the face of a real danger our first impulse should be to behave like little children. … We are afraid of being afraid. (Glover, 1940: 21–2)

The call for a ‘psychological ARP (Air Raid Precautions)’ was nurtured in the year following the outbreak of war, with articles appearing in medical journals predicting the scale of panic and spelling out the preventive measures required. These met with a measure of institutional success: an Emergency Mental Health committee was constituted, and wards in a few London hospitals were readied to take in civilian psychological casualties. However, when the German bombing campaign began in earnest in the summer of 1940, despite some early reports of panic, observers quickly shifted gears: from a discourse of nervous anticipation came the wearied noting of ‘monotonous’ reports from the psychological front line on the absence of bomb neurosis. Emergency stations for mental casualties folded up, and Glover and his colleagues turned to reflect on the remarkable capacity for human adaptation in times of tension (Glover, 1942: 28). 

This shift is recognized in the historical literature: indeed it became codified as early as Richard Titmuss’s landmark official history of the home front, *The Problems of Social Policy* (1950). There was no mass breakdown – in fact mental health improved due to wartime conditions of equality, stable employment, a renewed sense of responsibility to family and country, and officially sanctioned safety valves like evacuation. Titmuss’s judgement has in turn formed a central plank of ‘myth of blitz’ which, revisionist challenges notwithstanding, confirms the absence of civilian panic as a significant feature of Britain’s wartime experience (Calder, 1991; Mackay, 2003). 

Several explanations exist in the historical literature that seek to account for this shift, including the realist account (people simply did not panic), the propagandizing account (people did panic but were ignored), and the displacement account (people did not break down but did suffer less spectacular psychological injury). Here I do not contribute a new explanation, nor do I argue for one of the existing explanations. Instead, I look at how fear was turned into a practicable investigative object, how it was invested with significance at the level of science and of public policy, and how these considerations contributed to the production of the type of data upon which contemporary and historical judgement about the trajectory from nervousness to nerves is founded. To do this I focus on a single historical actor, Solly Zuckerman, and on his early war work for the Ministry of Home Security-funded Extra Mural Unit based in Oxford’s Department of Anatomy (OEMU). I will examine the process by which he forged a working relationship with fear in the inter-war period, and how he translated this work to questions of home front anxiety in his role as an operational research officer. In doing so I demonstrate the persistent *work* applied to the problem of civilian fear: by highlighting it as an ongoing research project, and suggesting links between seemingly disparate research objects, I wish to show how civilian ‘nerve’ emerged from within a particular analytical and operational matrix which itself had complex foundations. 

## Zuckerman’s apes

Solly Zuckerman was a South African medical student with a special interest in anatomy and primate behaviour, who travelled to London in 1925 to study at University College’s Medical School. There he met its head, Grafton Elliot Smith, also a leading diffusionist anthropologist and the foremost authority on the evolutionary aspects of brain morphology. Elliot Smith encouraged Zuckerman’s interest in comparative anatomy and psychology, and, after taking his MD in 1928, Zuckerman was appointed prosector at the London Zoo – a job whose principal responsibility was to determine the cause of the (numerous) deaths among the zoo’s primate inhabitants. Following a brief period at Robert Yerkes’s Anthropoid Experimental Station in the United States, Zuckerman returned to the UK in 1934 to take up an appointment as demonstrator at Oxford’s Department of Human Anatomy. 

Zuckerman’s interests neatly dovetailed with psychological, anthropological and sociological synergies developing in the 1920s and the early 1930s, and by the time of the publication of his most noteworthy pre-war work, *Social Life of Monkeys and Apes *(1932), Zuckerman was already well known to this interdisciplinary world. In the book’s preface Zuckerman made clear his perspective on the study of primate mind and society: ‘I have approached the subject from the deterministic point of view of the physiologist, treating overt behaviour as the result or expression of physiological events which have been made obvious through experimental analysis’ (Zuckerman, 1932: xi–xii). Zuckerman cast his approach in strict opposition to the ‘anecdotalism’ of 19th-century comparative psychology, its tendency to speculative, introspective and unsystematic anthropomorphism that made it little more than a ‘trail of fantasy’ (ibid.: 8). In his quest to neutralize fantasy, Zuckerman looked to the power of numbers as an essential tool: ‘Scientific thought’, he observed at the start of his follow-up monograph, ‘becomes increasingly difficult the less its material is amenable to quantitative treatment and the more it is related to deeply rooted emotional attitudes’ (Zuckerman, 1933: 1).

Though setting himself squarely against the perils of loose speculation, he also rejected a reductionist account of behaviourism that he associated with Watson and Pavlov, which to him made simplistic links between human and animal investigations, ignoring the selective and constructive character of perception and memory, and the cultural context in which these operated. ‘The effective stimuli involved in the behaviour of animals’, he maintained, ‘are mainly inherent in immediate physical events, which are in no way the by-products of the activities of pre-existing animals of the same species. Man, on the other hand, amasses experience through speech, and the effective stimuli underlying human behaviour are largely products of the lives of pre-existing people.’ Language and collective memory, in short, were the defining and distinctive features of human social activity. Despite this gulf, Zuckerman entertained a future in which a disciplined comparative psychology might provide a foundation for understanding human behaviour: ‘Cultural phenomena may not, in the last resort, prove to be absolutely different from physiological events’ (Zuckerman, 1932: 19). 

I only have space here for a brief synopsis of *Social Life*’s conclusions: the primary feature distinguishing non-human primate society from lower animals derived from the constant sexual availability of the female, leading to distinctive ‘ownership’ patterns – patterns that Zuckerman ultimately located in aggression and fear. As one enthusiastic reviewer put it: ‘The main conclusions may best be stated in a quotation: “Monkey society is based on dominance. The strongest monkey gets the most food, and the best of everything. The strongest male gets the most wives. Fear rules the monkey world …’’’ (Bernard and Bernard, 1934: 307). But how was fear to be understood scientifically? Not, for Zuckerman, at the level of an interior psychological self – which invited the twin dangers of anthropomorphism and anecdotalism – but instead as overt functional behaviour.^1^

*Social Life* was well received, widely and favourably reviewed in psychological, anthropological and sociological journals. Though Zuckerman was cautious about the application of his monkey research to human questions, others were less reticent: a reviewer for *Man*, for example, looked forward to the day ‘when we can observe the social behaviour of man by the same scrupulous techniques as that employed by Dr Zuckerman’ (Fortes, 1932: 168–9). Following *Social Life*, Zuckerman pursued his primate research, and also branched out into one of the most dynamic fields of the day – endocrinology, focusing in particular on male and female sex hormones. His research profile thus positioned him at the intersection of debates about the nature of human instinct, the limits of behavioural adaptability, the role of physiology and of culture in the expression of instinctual behaviour, and the place of primary emotions like fear in social organization. By the end of the 1930s Zuckerman counted as his correspondents a cross-disciplinary constellation of leading intellectuals including Bronislaw Malinowski, Julian Huxley, Frederick Bartlett and (briefly) Sigmund Freud. 

Another significant aspect of Zuckerman’s activities in the 1930s involved what we would now call ‘public engagement of science’, notably as a leading member of the ‘Tots and Quots’ dining group. It was the crystallographer and fellow ‘Tots’ luminary, J. D. Bernal, who led Zuckerman into war-related research. At the outbreak of war Bernal had been seconded by the Ministry of Home Security’s Research Department to study physical effects of bombing on buildings, and Bernal proposed that Zuckerman, with his anatomical expertise, be charged with extending the inquiry to the human realm.

## Demystifying blast

Zuckerman began his war work by searching for experimental methods by which to assess the physical effects of bomb blast on the human frame. As he reviewed the existing literature, however, he soon detected the sort of speculative anecdotalism that he had encountered in animal sociology. He also identified its root cause: the literature on blast was blighted by a lingering legacy of shell shock. The long-standing and complex debates about the physical and psychogenic aetiologies of shell shock need no detailed retelling here. Suffice to point out that one of the perennial questions confronting shell shock doctors in the war, and subsequent shell shock administrators, was the relationship between, in Frederick Mott’s terms, its ‘commotional’ and ‘emotional’ components. The mysterious element of shell shock, one that preoccupied practitioners and commentators alike, stemmed from its capacity to visit its damaging effects without leaving obvious signs of physical trauma. The title of one of Mott’s early lectures on shell shock is indicative of this interest: delivering a lecture entitled ‘Shell Shock without Visible Signs of Injury’ to the Royal Society of Medicine, he ventured that in such cases psychic trauma might play a significant role in what otherwise was a primarily commotional condition. Certain symptoms, Mott observed, ‘cannot be explained by cerebral commotion caused by the dynamic force generated by the explosive in a definite anatomical region of the brain, but must be associated with emotional shock caused by terror …’ (Mott, 1916a: xx). 

At his subsequent Lettsomian Lecture Mott related further mystifying accounts of the human encounter with high explosive charge. From the start of the war, Mott reminded his audience, journalists had reported on the strange phenomenon of instantaneous death among some soldiers exposed to shell fire, quoting from Ashmead Bartlett’s canonical account of the Gallipoli battlefield:

In one corner seven Turks, with their rifles across their knees, are sitting together. One man has his arm around the neck of his friend and a smile on his face as if they had been cracking a joke when death overwhelmed them. All now have the appearance of being merely asleep; for of the seven I only see one who shows any outward injury.

‘How’, Mott asked, ‘can we explain death without apparent bodily injury yet so instantaneous as to fix them in the life-like positions and attitudes thus realistically described?’ (Mott, 1916b: 336). 

Such haunting images of occult deaths continued to inform discussions about shell shock in the inter-war period, but they also framed a newly developing field of research devoted to the physics and physiology of what increasingly came to be referred to as ‘blast’ effects. The base-line assumption for blast researchers was that a single post-mortem sign at times found upon victims – blood-stained fluid around the mouth and nose – indicated traumatic haemorrhage of the pulmonary vascular system. But though researchers had agreed on this explanation, they remained divided as to the mechanism by which these injuries were inflicted. 

By the end of the 1930s there were three competing explanatory models. David Dale Logan of the Royal Army Medical Corps proposed that the suction component of the blast wave acted through the respiratory passages to lower alveolar pressure, resulting in capillary rupture (Logan, 1939). On the other hand, the Cambridge physiologist Sir Joseph Barcroft asserted that the positive pressure phase acted through the respiratory tract to increase alveolar pressure, causing their distension and rupture (‘Blast Injuries’, 1941: 89). The final theory, associated with the American physiologist David Hooker, held that lung lesions were a result of direct trauma to the body wall due to the impact of the blast’s pressure wave (Hooker, 1924). Unlike the theories proposed by Logan and Barcroft, which seemed to suggest a lethal dynamic peculiar to blast, Hooker’s explanation was more straightforward – blast was merely another way of delivering an old-fashioned blow to the body. 

Zuckerman was sympathetic to Hooker’s simplifying account, but found it grounded in mere conjecture. His task, then, would be to complete, through experimental demonstration, the demystification of blast. Zuckerman laid out his agenda in a 1939 ‘Memorandum on Concussion, Shock and Allied Conditions’, a draft document which also makes clear why he considered this an important area for research. First, since the home front faced significant exposure to blast, public understanding of its effects would be important for averting panic. Instead of rational understanding, however, Zuckerman identified in the contemporary literature precisely the opposite tendency – a fear-inducing discourse grounded in rumour and anecdote generated not only by popular writers like John Langdon-Davies (1938) but publicly recognized scientists and medics such as J. B. S. Haldane (1938) and Lord Horder (1939), and leading politicians, most notably Stanley Baldwin with his oft-cited warning that ‘the bomber would always get through’ (Baldwin, 1932). 

**Figure 1.**
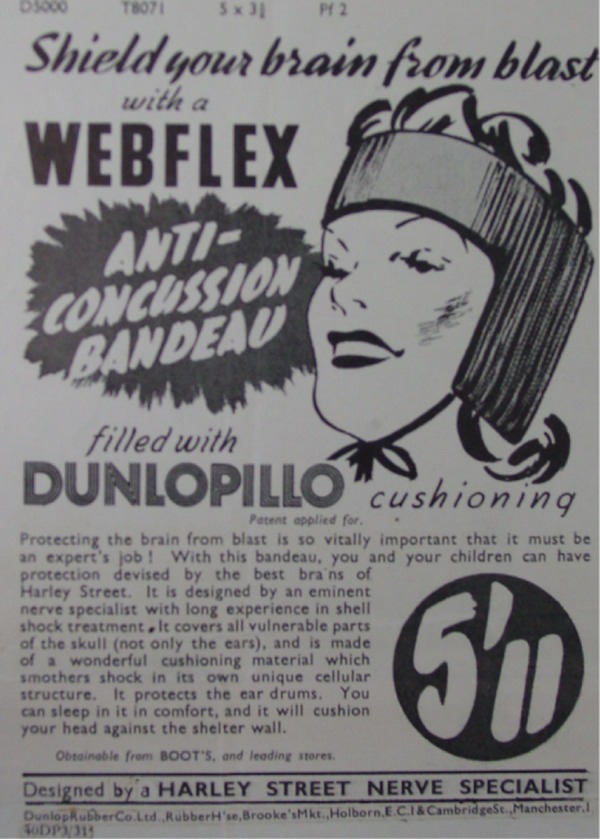
Public blast: from *Punch*, 16 October 1940; (SZ/OEMU/4/7/2), Zuckerman Archive, University of East Anglia.

For Zuckerman, then, blast was a constitutive component of the febrile state of public mind, in which an absence of science fed speculation – the ‘confused and largely anecdotal’ literature on blast, he lamented, ‘accounts for its great popular interest’ (‘Memorandum’, *c.*1939: 7). Confusion, moreover, had begun to be reified in the public domain: an ARP manual issued in early 1940, for example, advised that ‘the mouth should be kept open in order to protect the lungs from blast, and for this purpose it is useful to grip a piece of wood or of rubber tightly between the teeth’ (Anon., 1940: 992). When Zuckerman investigated the rationale for this instruction his fears were confirmed: the advice, according to Audrey Russell of the International Refugee Service, grew out of anecdotal experience of the Spanish Civil War: ‘The basis for these instructions’, Russell wrote, ‘was about as vague as bases usually are, but one gathered that keeping the mouth open allowed the breath to escape from the lungs and the pushing in of the chest wall absorbed a certain amount of the force of the blast – like giving with your hands on catching a hard cricket ball’ (Russell to Zuckerman, 1940). Zuckerman’s correspondence with the Dunlop Rubber Company provides a further example. A company representative wrote to Zuckerman in September 1940 seeking endorsement for its new ‘Anti-Concussion Bandeau’, but instead received a characteristically curt response demanding evidence that ‘the brain is affected by what is technically described as blast’ (Zuckerman to Dunlop Rubber Co., 1940). (See Figure 1.)

Against this undisciplined blast literature, and the broader public disquiet that it encouraged, Zuckerman positioned his laboratory as a sword of truth. As in his earlier battles with ignorance, ‘experimentally determined facts’, quantitatively expressed, would be the means of containing the emotive element of blast – both at the level of physiological explanation and at the level of public affect (‘Memorandum’, *c.*1939: 1). He made his target clear in his first report to the Ministry of Home Security, explicitly situating his nascent research programme as a response to generalized anxiety:

It will be remembered that the main point of medical interest in the problem of blast is the knowledge that men are sometimes found dead within the effective zone of an explosion without apparent wounds, and only showing bloodstained fluid trickling from the mouth or nose. … These fatalities are usually ascribed to the decompression component of the blast wave. This is a view generally put, without evidence, in the clinical literature. (‘First Report’, 1940: 1)

**Figure 2.**
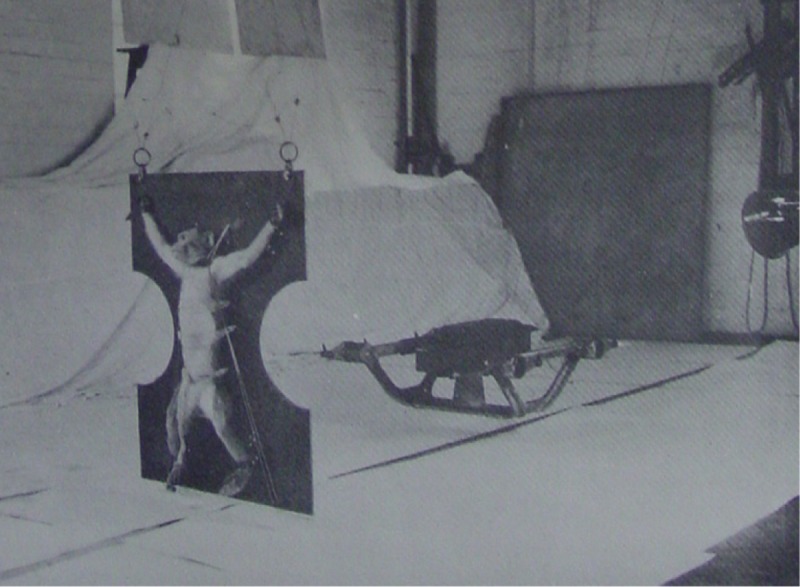
Experimental blast: (SZ/OEMU/3/1), Zuckerman Archive, University of East Anglia.

The first step in redressing this situation was to resort to the disciplined space of the lab. In the ‘wild’, bomb sites were chaotic and emotionally charged, resistant to analysis. To render blast more legible, Zuckerman first sought to establish a base-line for human and animal tolerance. Through a graded series of controlled explosions, and the measurement of incremental bodily effect at determined distances from blast of varied intensity, ‘an approximate idea could be obtained of the minimum air-pressures which are likely to be dangerous to people, and a relative scale of injury could be estimated’ (‘Memorandum’, *c.* 1939: 16). (See Figure 2) 

This work initially contained a psychological component: alongside physical trauma, Zuckerman proposed to note changes in behaviour and any neurological signs indicative of damage to the brain and spinal cord. However, as Zuckerman explained in his first report, this broad physio-psychological investigative remit was altered in the course of the initial experiments by what he described as ‘unexpected findings leading to important conclusions’. Recalling the primary purpose of the experiments – to explain (and thus conceptually and operationally contain) occult deaths – he noted that previous commentators had focused on the brain and the lungs as the two bodily zones most implicated in the phenomenon. Zuckerman’s investigations convinced him of the insignificance of brain injury for ‘true blast’ pathology: the behaviour of his monkeys, rabbits and rats ‘appeared in no way disturbed; almost all rabbits ate grass offered, and monkeys perfectly normal’. Post-mortem evidence supported these findings: despite careful search, no lesions to the nervous system were found (‘First Report’, 1940: 2).

**Figure 3.**
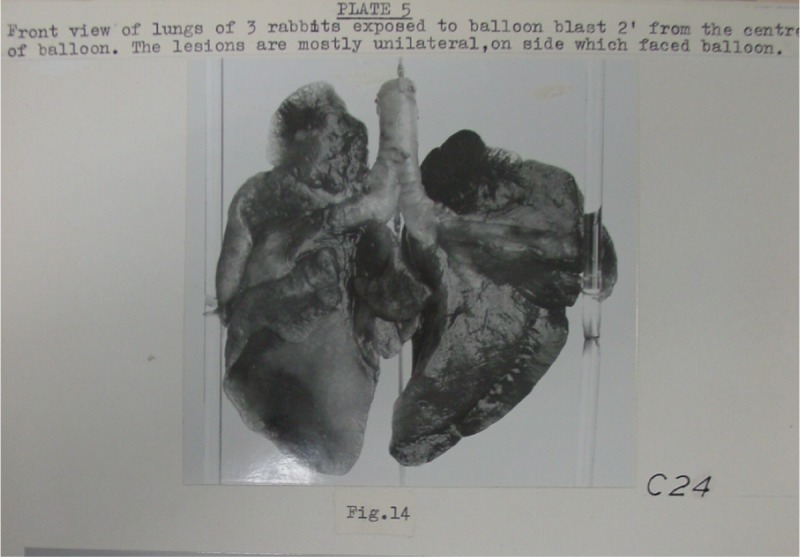
Pathology of blast: (SZ/OEMU/3/4/3), Zuckerman Archive, University of East Anglia.

The key to unravelling the mysteries of blast, these findings suggested, lay not in shattered nerves, but in traumatized lungs. Post-mortem attention to lungs, moreover, yielded a further significant result – lesions were found exclusively on the side of the animal that had faced the explosion. If suction or distension were the cause of trauma, Zuckerman reasoned, these would act on the entire respiratory tree, and thereby damage both lungs. ‘Presumably, when the pressure wave hits the body, the space between the chest wall and the heart and liver becomes suddenly reduced, and that part of the lung which normally fills this interval is violently compressed’ (‘First Report’, 1940: 14). Hooker was right: Zuckerman’s experiments had suggested an experimentally grounded framework for containing the spectre of blast, figuring it as straightforward external trauma rather than either as an assault on the brain (and by extension the ‘mind’), or as a dynamic force which insinuated itself into interior physiological processes. (See Figure 3) 

To secure this reading of blast injury, however, Zuckerman had to move out from the laboratory and back into the chaos of the bomb site. Making a success of this confirmatory stage, however, presumed a reliable flow of clinical and post-mortem information, and to secure this a circular, written on his behalf by the Emergency Medical Service director Francis Fraser, was sent to EMS hospital officers in June 1940 which focused attention on occult blast victims:

People are sometimes picked up dead within the effective zone of an explosion without any external signs of injury, and showing only bloodstained fluid in the mouth and nose. … Observations are urgently required on human air-raid casualties as to the incidence of these [sub-lethal pulmonary] lesions and these observations will probably form the basis for a scheme of diagnosis and treatment. (‘Effect of Explosion Blast’, 1940 [n.p.])

Within a few months Zuckerman was complaining that the field was not providing adequate information, and began recommending measures for the better collection of data. He called for a more structured system for post-mortem investigation, criticizing the sparse information provided by local pathologists ‘unlikely to have time available for proper investigation, nor likely to know much of the type of lesion to be looked for’ (‘General Observations’, 1940: 1). 

In correspondence with hospital practitioners eager to supply him with cases of blast, moreover, Zuckerman was often less than fulsome in his thanks, disputing their diagnoses which for him did not achieve the precision required for proper scientific knowledge. In correspondence with Bryan McSwiney, the St Thomas physiologist and member of the MRC’s Shock Committee, Zuckerman chastised him and his colleagues for supporting Barcroft’s suction theory of blast on the basis of post-mortem appearance alone: ‘I can’t for one second see how one could infer the mechanism of the injury from just looking at the lesions. That’s all that has been done so far, and in that method anyone’s guess is as good as any other’ (Zuckerman to McSwiney, 1940). 

A long-standing and at times tense correspondence with G. R. Osborn, pathologist at Derbyshire Royal Infirmary, again centred on Zuckerman’s insistence on the insufficiency of post-mortem evidence unsupported by experimental work. This exchange reached a wider audience when Osborn published accounts of blast cases in the *British Medical Journal *in 1941 (Osborn, 1941a). Zuckerman, in a letter to the editor, expressed scepticism on whether several of Osborne’s cases had ‘in fact much to do with the direct effects of blast – except in so far as [he] speculates about the subject. His cases reveal no evidence of direct blast injury as the term should be used’. Instead they suggested ‘more understandable forms of trauma due to secondary and tertiary effects of blast-violent displacement, falling masonry, and secondary missiles’. It was only those (in his view rare) cases in which these forms of violence were absent, Zuckerman insisted, that merited consideration as blast (Zuckerman, 1941a: 645). In turn, Osborn launched a critique of Zuckerman’s distorted standards stemming from his unhealthy lab fetish. He ridiculed Zuckerman’s use of pure blast physics to explain clinical findings that, for him, were better explained by ‘the human factor’, urging Zuckerman not to ‘detract from his valuable experimental work by making statements about humans which are almost nonsense’ (Osborn, 1941b: 869).

Faced with an ad hoc and, for him, imprecise information flow, Zuckerman pushed for a human equivalent to the recently established means for deriving data on damage sustained to Britain’s physical infrastructure – the Bomb Census, initiated by the Ministry of Home Security in September 1940: ‘The relationships between the bombs and the amount of structural damage done are already being carefully investigated’, he noted in an internal memo. ‘Similar relations between the bombs and the amount of human structural damage do not seem to be receiving attention’ (‘General Observations’, 1940: 1). Zuckerman prevailed, and in the winter of 1940 he took overall charge of the newly constituted ‘Casualty Survey’, comprised of a team of fieldworkers based at Guy’s Hospital and led by the young Thomas McKeown. 

**Figure 4.**
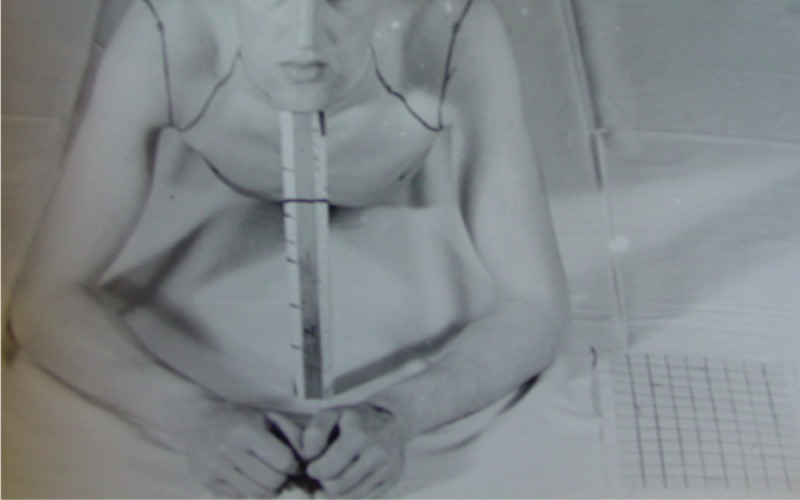
Mapping vulnerability: (SZ/OEMU/2/11/22), Zuckerman Archive, University of East Anglia.

The survey’s principal contribution to Zuckerman’s blast research was twofold. First, it enabled the collection of raw data from the field on bomb casualties in a reliable, standardized form. To do this Zuckerman’s team devised a series of field tools – the most striking of these being a ‘wound chart’ which, by dividing the body into standard regions, enabled both data comparability across incident scenes, and the calculation of surface exposure and vulnerability – the ‘mean projection area’ of the body and its parts (see Figure 4). Second, it brought interpretive order to the bomb site by short-circuiting the anecdotalism that had till then hampered scientific understanding. This was to be accomplished by the deployment of a series of analytical devices, including (following the suggestion of the noted medical statistician Austin Bradford Hill) the use of random sampling techniques that explicitly rejected unsystematic and subjective criteria. The physical and emotive complexities of a recent bomb site were further rendered analytically manageable by recourse to statistical abstraction: the ‘vulnerable area’. Derived with the help of Frank Yates and others at the Rothamsted Experimental Station, this concept enabled ‘a single measure of the powers of resistance to the effects of a given type of high explosive weapon possessed either by a given structure or by a person occupying a given position’, one that could be ‘expressed mathematically by choosing a system of horizontal Cartesian co-ordinates’ (David and Garwood, ‘Applications of Mathematical Statistics’, 1942: 1).

Through these instruments, Zuckerman sought to contain blast, as a patho-physiological problem and as a source of dangerous public anxiety. Within the space of a year he felt confident enough to declare victory on the first of these fronts. In a letter to the Nobel Prize-winning physiologist Edgar Adrian he reported that his experimental conclusions about the significance of lung damage had been confirmed in the field. ‘We have kept a close watch for cerebral concussion occurring as direct impact of blast wave on humans’, Zuckerman wrote, ‘but so far our Casualty survey failed to disclose an instance.’ It is clear, he concluded, ‘that the damage is simply a bruising of lung tissue due to the impact of the pressure wave on the chest wall’ (Zuckerman to Adrian, 1941). 

Zuckerman’s taming of blast did not go unnoticed. For the *Manchester Guardian*, Zuckerman’s research had revealed a surprising resilience of the human body compared with the nation’s physical infrastructure, leading to the conclusion that blast effects were ‘much less harmful than was previously thought’ (Anon., ‘ARP Research’, 1941: 5). Zuckerman’s strict definition of blast, moreover, might go some way to alleviate the second source of difficulty – public terror of what it called the ‘capricious effects of blast, which everyone has noticed’. For the *BMJ*, Zuckerman’s lessons applied equally to its readership, showing the need for ‘the greatest caution in labelling any given condition occurring in man exposed to air raids as resulting from “blast”’. By following his strict classificatory model, it concluded, ‘much unwise discussion would be prevented and possibly much needless suffering, both physical and psychological, stopped’ (Anon., ‘Blast Injuries’, 1941: 90). Zuckerman had thus laid the spectre of shell shock to rest: survivors of close-range, large-calibre shell explosions had been shown to be in reality suffering from lung damage. Moreover, the objective approach of his ‘exposure to risk’ fieldworkers, in the view of the *BMJ*, had provided the groundwork for a more balanced understanding of the dangers of air raids, the public dissemination of which ‘would act as a corrective to the natural tendency of all to magnify the size of the bomb and the nearness of themselves to it’ (ibid.: 90). Zuckerman’s programme of disciplining the public’s imagination, of forging the basis for rational civilian behaviour by displacing anecdote and mystery with rigorous experimental data and the analytics of risk calculation, was thus recognized as a means of transitioning from pre-war anxiety to wartime nerve. 

## Surveying neurosis

In August 1941, Zuckerman turned his attention explicitly to the question of mental effects in humans of exposure to air raids. The spectre of ‘civilian neurosis’, as discussed previously, had haunted pre-war commentators and officials. Glover’s 1940 BBC broadcast in some respects marked the culmination of years of rich speculation about the likely impact of total war on the civilian population. The noted psychoanalyst John Rickman, in an extended review of Langdon-Davies’s *Air Raid*, identified the physical characteristics of high explosive bombardment as presenting a novel threat to emotional stability:

People cannot endure the terrific percussion; they lose voluntary control and run hither and thither, aimlessly, fatuously, and it is some time before they become at all collected. There seems to be something specially disturbing in the combination of surprise and suspense with the whack of the new kind of explosive, far more disturbing than the raids that are expected of the ‘softer’ blow of the older type of bombardment. (Rickman, 1938a: 361)

Drawing on impressions gathered from Spain, he also noted the subjective dimension of raid panic, its lack of containment by any rational evaluative standard of danger: ‘the horror roused by air raids appears to be disproportional to the actual risk to any particular individual’ (Rickman, 1938a: 367). In a much-cited contribution to the *Lancet*, Rickman argued that this neurotic element needed to be combated with risk-based public instruction: ‘The public in fact should be instructed to think in terms of relative quantity, of more-or-less (reason, conscious mental action) rather than of all-or-none (blind belief, unconscious mental action)’ (Rickman, 1938b: 1295). 

Such discussions about psychological ARP, again, were significantly framed by the legacy of shell shock. As Ben Shephard and others have noted, the problem of compensatable civilian neurosis was a clear concern for pension officials in the lead-up to war (Shephard, 1999; Jones, Palmer and Wessely, 2002). The vexed question of direct physical impact and ‘emotional’ trauma was written into wartime legislation with the 1939 Personal Injuries (Emergency Provisions) Act, which stipulated that only cases of trauma with a demonstrable physical aetiology were to be considered for compensation. This policy was explained to practitioners in a circular issued following the passage of the Act: ‘The diagnosis of concussion should be made only when the history or clinical symptoms leave no reasonable doubt that the patient has suffered physical injury either by the direct explosion of a shell or bomb, by being knocked over by it, or by being buried under debris of a building or shelter’ (‘Diagnosis of Concussion’, 1939). Mere proximity to a blast explosion, ministry officials insisted, was insufficient to admit casualties to the scheme. 

The Act proved contentious, and in the first year of bombing several parliamentary questions were raised demanding explanations for refused compensation cases. Ministry responses tended to cite physical proximity as the determining measure – thus, for example, a Mrs Pearce was granted payment because ‘she was near enough to the explosion to have sustained the physical effects of blast’, whereas a railway guard suffering from shock as a result of bombs dropping near to his brake-van was denied payment when ‘careful inquiry’ revealed ‘that at the time in question he was 350 yards from the nearest bomb explosion and suffered no physical injury therefrom’. In an internal memo one ministry official acknowledged the difficulties faced when relying on proximity, observing that ‘it would be almost impossible to know where to draw the line in regard to proximity to the explosion. … There the position stands and I can see we are going to have a spot of trouble about it’ (Glover to Ministry of Pensions, 1940).^2^

The compensation question, and the need to contain civilian psychological responses to bombing, was a key factor in shaping Zuckerman’s programme for investigating civilian nerve – what would become known as the Hull–Birmingham Neurosis Survey. However, in the account of the survey’s origins provided in his autobiography, Zuckerman emphasized a second, allied consideration. The idea, he recalled, stemmed from a conversation in late August 1941 with Churchill’s chief scientific adviser, the physicist Frederick Lindemann (newly ennobled as Lord Cherwell). At this point in the course of the war there was a live debate about the purposes and powers of the RAF: some, led by Cherwell and Churchill, advocated area (or ‘tactical’) bombing of the civilian population of Germany with the objective of breaking civilian morale, while others urged a more limited, ‘strategically’ targeted use of air power against military and economic infrastructure. This debate had its roots in the immediate aftermath of the experience of relatively limited civilian bombing campaigns of the First World War, and especially in the influential and forcefully expressed position taken by Sir Hugh Trenchard, chief of the Air Staff from 1919 to 1930, on the capacity of air power to inflict telling psychological damage on a combatant’s home front. Trenchard grounded his argument in an attention-grabbing statistical ratio: that the ‘moral’ to ‘material’ effect of bombing stood in ‘a proportion of 20 to 1’ (cited in Biddle, 1995: 92).

The doctrine’s ostensible numerical solidity, historians agree, was wholly illusory – Trenchard was, according to Malcolm Cooper, ‘master of the wholly unfounded statistic’, his declaration resting on what Titmuss dismissed as ‘instinctive opinion’ (Biddle, 1995: n. 6 and n. 50). Yet as Webster and Frankland observe, Trenchard’s ratio ‘continually’ featured in high level discussions on the use of air power in the 1930s and into the early years of the war. It was in this context that Cherwell, according to Zuckerman, sought an improved ‘objective basis’ for area bombing, and it was this that prompted Zuckerman to propose a survey of the overall effects of bombing on selected English cities. ‘I suggested that we choose for study Hull and Birmingham’, he continued, ‘first because the Bomb Census had an almost complete tally of the bombs that had fallen on them, and second because they could be regarded as typical of manufacturing and port towns’ (Zuckerman, 1978: 140).

Zuckerman’s research into civilian neurosis, then, was shaped by two powerful contemporary interests, both of which centred on what he would readily recognize as empirically dubious maxims inherited from the First World War and nurtured in the febrile atmosphere of the 1930s. Attention to the survey’s archival record suggests that both left their mark on the survey’s design and findings, and that Zuckerman negotiated these interests in ways that drew upon the concerns and techniques of Zuckerman’s previous war research. 

The first mention of such an undertaking in the Zuckerman archive is found in a memo entitled ‘First Note on Proposed Psychological Survey’ and dated in Zuckerman’s hand ‘about August 12, 1941’ (presumably before Zuckerman’s ‘late August’ meeting with Cherwell). This memo starts with a section outlining the purposes of such a survey: ‘(a) to study the immediate reactions of members of the general public who have actually been exposed to risk during the course of raids, and (b) to obtain information about shelter behaviour, and (c) to study the behaviour of Civil Defence personnel who are exposed to risk over a considerable period’. ‘The general problem’, it continued, ‘is closely related to that of the maintenance of morale’ – trained observers might be able to define signs of civilian unrest, which in turn would inform official policy and propaganda (Zuckerman, ‘First Note’, 1941: 1).

In justifying his survey, Zuckerman sought to distance it from contemporary ‘morale’ investigations conducted by ‘amateur organisations’, trading in ‘anecdotal information’ – Zuckerman’s long-standing *bête noire* – and producing ‘highly suspect and coloured’ information. His quest for a framework for generating firm empirical data on the state of the civilian mind was driven by a second, strategic imperative – the need to by-pass potentially powerful opposition from the Ministry of Pensions. ‘A general enquiry into the behaviour of people in raids’, Zuckerman warned, risked falling foul of the ministry, due to its view that ‘enquiries into neuroses breed neuroses – and neuroses can become a considerable burden on the public exchequer’ (Zuckerman, ‘First Note’, 1941: 2). 

It was vital to circumvent ministry suspicion, Zuckerman argued, not merely in the interest of home security, but to capitalize on a unique set of experimental conditions: ‘The opportunity that is presented by raids for enquiry into basal patterns of human behaviour’, he urged, ‘is one that we hope will not present itself after this war and one which should be seized to-day.’ To achieve its aims without provoking official opposition, the survey had to be framed not as a ‘psychiatric enquiry but as an enquiry into behaviour’. If properly conducted, he continued, ‘no one should get impression that neuroses are a natural outcome of the conditions imposed by air-raids’ (‘First Note’, 1941: 2). It was to his already established Casualty Survey that Zuckerman turned to ensure the requisite investigative discipline: as a supplement to their assessment of physical damage, his ‘exposure to risk assessors’ could make enquiries ‘into behaviour of all concerned in chosen isolated incidents, chosen at random’, assisted by a ‘trained observer who can indicate to the rest of the team what observations to make, and which observations they make are of significance’ (ibid.: 3). 

Several interesting themes were developed in subsequent documents. First, there was the importance of the (official and public) fear of fear problematic, and the way it informed Zuckerman’s approach to and justification of the survey. In his initial letter to the Ministry of Pensions’ chief scientific adviser, Zuckerman was at pains to emphasize both the utilitarian motives and the scientific parameters of his proposed study: ‘My department is engaged on a survey of the exact circumstances in which people get injured in air-raids’, he began. ‘This enquiry has hitherto concerned itself only with the question of physical injury. The results have been of great value in the evaluation of shelter policy.’ His proposal to extend the survey arose from the concern that ‘in overlooking the psychological aspect I may miss information of paramount importance in the problems of shelter policy’. Zuckerman categorically distinguished his proposed survey from ‘undisciplined psychiatric studies’. ‘Leading questions about psychological disturbances’, he insisted, ‘would not be put to the subject of our inquiry; in studying a particular incident what we should aim at would be to obtain an objective statement from those involved about what they thought happened’ (Zuckerman to Ministry of Pensions, 1941: 1).^3^

Contextual constraints on official neurosis surveys, then, neatly dovetailed with Zuckerman’s prior commitment to disciplined enquiry into objectively measureable indices of emotion (and an avoidance of speculations into internal mental states), and his broader aim to counter a priori alarmism generated by anecdote and public ignorance. It is not surprising, then, that Zuckerman’s carefully framed proposal was given the green light, and that once approved Zuckerman proceeded to subsume it within the existing disciplines of his Casualty Survey, by grafting questions about mental stress onto the operational procedures in place for investigating physical stress. 

By war’s end, Hull had attained the unenviable position as the second most heavily bombed city in Britain. When Zuckerman’s survey commenced, roughly half of its housing stock had already suffered damage in a succession of raids between March and November, 1941, including a handful of especially intense attacks over four nights in April, May and July. It was these raids that Zuckerman selected for investigation. On the advice of the influential Maudsley psychiatrist Aubrey Lewis, Zuckerman appointed Russell Fraser, a New Zealander working under Lewis at the Mill Hill Emergency Hospital, to serve alongside his existing casualty surveyors as psychiatric adviser. Though the survey incorporated material from both Hull and Birmingham, it was Hull that emerged as the focus of Fraser’s neurosis research. Dock workers were the principal investigative target, approximately 900 of whom were selected – following the methods of the Casualty Survey – on a random sample basis for interview between November 1941 and January 1942. 

On appointing Fraser, Zuckerman made it clear that he would have to operate within the research parameters of the Casualty Survey, a point reinforced by Zuckerman’s summary dismissal of an attempt by Fraser to assert greater autonomy for his element of the inquiry (Zuckerman to Fraser, 1941). Fraser appears to have accepted his subordinate role, and in consultation with Zuckerman he devised a means of determining the existence of raid-induced neurosis that depended primarily on tangible behavioural indices. Drawing on his experience at Mill Hill, Fraser proposed in a letter to Zuckerman a predominantly behaviouralist measure of neurosis. Preliminary assessment of a subject’s ‘constitution’ through past health experience, of changes in habits – e.g. in sleep patterns, alcohol and tobacco usage – would enable a rough classification according to ‘personality’.^4^ Following this preliminary personality assessment, Fraser sought to evaluate the subject’s current ‘raid state’, which he proposed to derive through a stimulus–response correlation. As raids took place in the night, those who manifested abnormal levels of anxiety during the day could be classed as neurotic. For those disturbed only at night, Fraser proposed to determine whether this might be classed as ‘excessive’ on similar grounds:

Excessive anxiety is graded according to various points: i) Where there is evidence of increased sensitivity (i.e. greater ease of development of anxiety since start of bombing); ii) The extent of the somatic symptoms, the type of stimulus that brings them on, and the duration for which they last. (Fraser to Zuckerman, 1941)

Fraser’s framework for recognizing neurosis as a function of externally identifiable behaviour was reinforced in a subsequent memo, which again emphasized the need to determine changes in habits, bodily symptoms, and the degree to which raid reaction was commensurate to external stimulus. This enabled classification that by-passed subjective accounts of internal states:

Individuals were not asked to describe the degree of their fear; but rather to describe symptoms which it was implied that everyone felt; and the impression was given that differences were expected to indicate features of their physical constitution. By this procedure it is felt that reliable answers were usually obtained. (‘Criteria for the Diagnosis of Neurosis’, undated)

Through this investigative matrix, civilian neurosis – object of pre-war speculation and hyperbole – was recast as another front in Zuckerman’s wider war on (undisciplined) fear. 

**Figure 5.**
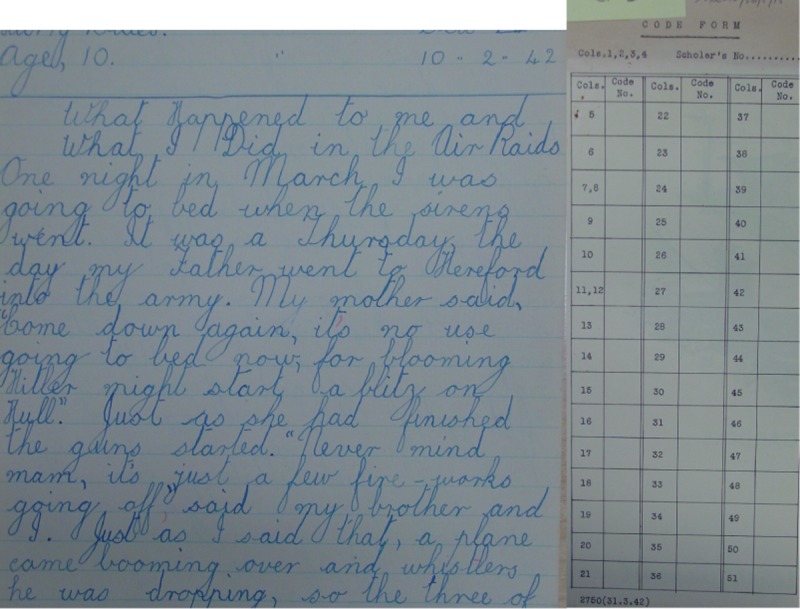
Coding fear: (SZ/OEMU/56/8/15), Zuckerman Archive, University of East Anglia.

The survey’s objectifying approach to civilian emotional states can be appreciated from another angle, by examining its proposed treatment of one of the more intriguing set of materials generated by the Hull survey: a trove of commissioned essays from Hull school-children on their air raid experiences. At face value, the gathering of stories from hundreds of vulnerable adolescents suggests a relaxation of his injunction against interiority, an invitation to engage in realms of fantasy. However, though the vast majority of the essays received ultimately lay unread, Zuckerman’s framing of the essay title and his methodology for the essays’ analysis reconfirmed rather than violated his overall investigative parameters. The pupils were set a question designed to maintain the survey’s focus on overt behaviour: ‘What I Did in the Air Raids and What Happened to Me’. Nor were they to be read in search of emotional insight. Instead, Zuckerman, along with Bradford Hill, prepared numerous drafts of a reading key that would facilitate the identification and recording of more prosaic information. In the final version of this document, readers were presented with a form (see Figure 5) containing some 50 columns, in which they were to record, with reference to a prepared numerical key, instances in which the essays made reference to specific features of raid experience. Just over 30 of these were arranged under the heading ‘Behaviour During Raid’, with information to be gleaned under this heading including the position of the writer during the raid, severity of experience, injury and damage to bodies and buildings, and statements about air raid precautions (shelter conditions, warden behaviour, etc.). Of these entries, only four addressed the emotional register (‘Analysis of Raid Essays’, 1942). The emphasis on objective, external behavioural indices as opposed to anecdote and interior sensibility was confirmed by the technology designed for processing the essays. Fragmenting individuated stories as discrete numerical values tabulated on code cards would ensure their containment as a collective synthesis of common behaviour, rather than a record of singular affect (Hull School Essay series, 1942). 

## The uses of Hull

It is not as a study of neurosis that the Hull survey made its biggest impact. As Zuckerman acknowledged at the time of its design and implementation, and as the correspondence and draft reports confirm, it was something of a rush job, as the participants struggled to establish settled investigative parameters and to fix the human and material variables into a workable experimental model. Individual and aggregate behaviour in the midst of air raid chaos was difficult to plot neatly onto a normal/abnormal scale. Assumptions about the prior mental status of the survey’s over 900 subjects were heavily dependent on their own subjective accounts of past symptoms and behaviour, and Fraser’s criteria for classing the degree of neurosis present in each subject were acknowledged as somewhat crude. Time and logistical constraints also led to numerous fruitless lines of inquiry, most obviously the aborted analysis of the schoolchildren’s essays on their raid experiences.

Nevertheless, in his final report on the combined results of the Hull and Birmingham survey, co-authored by Bernal, Zuckerman was clear in his overall assessment of the lesson to be drawn from the survey. Among the summary of conclusions listed at the front of the document is the following emphatic statement: ‘THERE IS NO EVIDENCE OF BREAKDOWN OF MORALE FOR THE INTENSITIES OF THE RAIDS EXPERIENCED BY HULL OR BIRMINGHAM’, a point developed in the body of the report:

In neither town was there any evidence of panic resulting either from a series of raids or from a single raid. The situation in Hull has been somewhat obscured, from this point of view, by the occurrence of trekking, which was much publicized as a sign of breaking morale, but which in fact can be fairly regarded as a considered response to the situation. In both towns, actual raids were, of course, associated with a degree of alarm and anxiety, which cannot in the circumstances be regarded as abnormal, and which in no instance was sufficient to provoke mass anti-social behaviour. There was no measurable effect on the health of either town. (Zuckerman and Bernal, ‘Quantitative Study’, 1942e: 3)

The reference to trekking, sandwiched between clear statements about the absence of panic, anti-social behaviour and ill-health, is a telling one. The Hull ‘trekkers’ – those members of the civilian population who made nightly journeys outside the city to escape raids – had in prior months been vilified in the media and by officials as the embodiment of dangerously weak civic morale. His conclusion represented another victory in his war on fear: trekking as a presumed category of wartime neurosis, like so much else in the emotional landscape painted by unscientific speculators, was exposed as unfounded anecdote. 

The Hull survey, then, fitted easily within the growing contemporary consensus that pre-war predictions about an epidemic of raid-induced panic had proven groundless. For Zuckerman, moreover, it complemented his prior analytical containment of the consequences of bombing: the effects of total war were neither materially nor mentally as devastating as had been feared. The results of his research, he suggested, should ‘prove reassuring’ to the public mind made anxious by loose speculation (Zuckerman, draft letter, 1942). Yet it was not as an exercise in rational reassurance that the Hull survey is best known. Indeed, quite the opposite – historical interest in the report has focused on its subsequent use by Lord Cherwell in his highly controversial and contested efforts to use area bombing to break German morale. For Cherwell, the main interest of the report lay not in the niceties of classifying degrees of neurosis but in a simple arithmetical ratio of tonnage and intensity to human and material strain. If the civilian population had suffered significant mental breakdown, he wanted to know, what were the characteristics of the attacks that had achieved this effect? If not, how much more, and what kind of attacks, would produce this outcome? 

The survey’s final report clearly indicates that, despite its initial framing as an assessment of the state of domestic mental resilience, Cherwell’s agenda had ultimately taken priority. In stark contrast to the preliminary drafts produced between January and April 1942, which foregrounded the sociological and psychological work of the survey, the final version issued on 8 April emphasized the data generated on the physical scale of the German attacks – tonnage dropped, area intensity, and the corresponding human and material casualties that had resulted. Though this information was implicitly correlated with an assessment of the impact on civilian morale, no more detail was provided than the emphatic statements cited above about the absence of breakdown and panic. 

Instead, it was Cherwell’s interest in using the data to derive a morale-breaking bombing calculus that took precedent, as indicated by the report’s final sentence: ‘We are not yet in a position to state what intensity of raiding would result in the complete breakdown of the life and work of a town, but it is probably of the order of 5 times greater than any that has been experienced in this country up till now’ (Zuckerman and Bernal, ‘Quantitative Study’, 1942e: 6). Hardly a statement of arithmetical certainty, its provisionality – and its problematic status for its authors – is underscored by the fact that in the numerous drafts leading up to the final report the sentence appears verbatim, but with a blank space where the number was to be entered (Zuckerman and Bernal, ‘Quantitative Study’, 1942c and 1942d). Subsequent correspondence indicates Zuckerman’s continued reluctance to stand by this quantitative approach to civilian morale as a definitive statement of empirical fact – in response to a question about the relative efficacy of large or small bombs for delivering a morale-breaking raid, for example, Zuckerman reported that a review of his files yielded ‘no real facts on which to base any definite conclusion, though there are plenty of opinions’ (Zuckerman to J. G. Richards, 1943).

Yet of course it was this ratio that was of most interest – and use – to those engaged in the strategic bombing debate. In a minute prepared for Churchill on 30 March, some nine days in advance of the publication of the final report, Cherwell advised that the survey’s findings supported the policy of targeting German civilian morale. Though actual infrastructural damage suffered by Hull had been modest, he wrote, ‘signs of strain were evident’. If Bomber Command were to conduct raids on German cities on an intensified scale, he concluded, ‘there seems to be little doubt that this would break the spirit of the people’ (Cherwell minute, 1942, in Zuckerman, 1978: 142). 

This conclusion – which Zuckerman insisted in his autobiography ‘was the very reverse of what we had stated’ (Zuckerman, 1978: 146) – has featured prominently in historical accounts of Britain’s air campaign. There is widespread agreement that it intervened at a crucial moment in the debate about the value of prioritizing the destruction of civilian morale through area bombing, though whether it was a ‘decisive’ intervention remains a matter of debate (e.g. Webster and Frankland, 1961: 337; Kirby and Capey, 1997: 665). For Zuckerman, by reinvigorating inter-war hyperbole about the powers of bombing to induce crippling fear in the civilian population, the ‘misuse’ of the Hull survey represented a clear setback in his campaign to use science to slay myth. Yet this was not a defeat that Zuckerman would easily accept, as is amply demonstrated by his later war work and, arguably, beyond. 

By early 1943 Zuckerman had been appointed scientific adviser to the commander-in-chief of the North African and Mediterranean Air Command, Arthur Tedder, for whom he conducted further detailed empirical research on the comparative effectiveness of different uses of air power. Zuckerman’s reports, filled with vulnerability indexes and allied calculations, consistently emphasized the value of selective, strategic targeting bombing of transport nodes rather than wasteful and ineffective attempts to crush morale through area bombing. Supported by Tedder’s powerful patronage, Zuckerman took active part in planning a number of the command’s highly successful and celebrated operations featuring the selective use of air power. In January 1944 Zuckerman returned to the UK to provide advice on the preparatory bombing strategy for the D-Day offensive, where again he laid out the scientific case for strategic over area bombing. At the war’s end Zuckerman was given the directorship of the British Bombing Survey Unit (BBSU), which was set up to assess the effectiveness of the air offensive against Germany. The BBSU’s final report, issued in 1946, provided Zuckerman with a high profile forum for laying the exaggerated inter-war discourse of civilian vulnerability to rest, concluding that the misguided emphasis on undermining civilian morale had unnecessarily prolonged the war. 

The lessons of Hull can also be detected well beyond the war years, most directly in his criticism of subsequent bombing campaigns that pursued the chimerical objective of subduing civilian morale, such as the US campaign in South-East Asia (Zuckerman, 1978: 148). But as Richard Maguire has recently argued, Zuckerman took his fight to further fields, notably in the controversial position he adopted on Britain’s post-war nuclear arms programme. For Zuckerman, the policy of successive governments to pursue ever more powerful nuclear weaponry was grounded in the emotive power of conventional thinking transmitted through successive generations, untested by empirically based rational calculation. Military and civilian policy-makers alike, he complained in terms redolent of his earlier battles against fear-generating myth forged in anecdote and unchallenged by exacting empirical testing, ‘are impelled along a road paved with outworn nuclear convictions, and with dogma enshrined in fading secret memoranda’ (Zuckerman, cited in Maguire, 2007: 132). Zuckerman’s war was protracted, determined and fought on many fronts. In this war, moreover, his faith in the power of quantitatively expressed empirical investigation at times failed to meet his objectives. Numbers, indeed, could themselves serve the purposes of myth-makers.

## Notes

1. For more on Zuckerman’s account of the dynamics of monkey fear/dominance/sex system, see Haraway (1978); Burt (2006) covers Zuckerman’s experimental work more generally, including aspects discussed in this article.

2. The issue of compensation for ‘emotional’ injury, of course, had a long history in the Anglo-American legal system, and the traditional view held that fear without impact was non-compensatable. But, in his review of recent case law, Winston Smith observed that the requirement of physical impact was being eroded, tellingly citing the example of blast to illustrate the ‘meaningless’ threshold of physical impact with physical object: ‘Waves of air from an explosion may cause “blast injury” or traumatic sounds. Waves of ether may cause visual injury or burns. These involve “physical contact” as truly as striking plaintiff with a weapon’ (Smith, 1944: 304–5).

3. In an internal memo, Zuckerman underscored the need to present the survey in these limited terms: ‘Whatever is done, it is highly desirable that our enquiry should not be regarded as a psychiatric enquiry but as an enquiry into behaviour.’ If it was properly framed, he concluded, no one should get the impression that ‘neuroses are a natural outcome of the conditions imposed by air-raids’ (‘First Note’, 1941: 2).

4. It is worth noting that for this personality assessment, Fraser’s case notes revealed his use in some instances of a controversial physical mapping methodology drawn from the German ‘characterology’ tradition – specifically that of Ernst Kretschmer. Kretschmer’s correlation of physical morphology onto psychological types was the subject of ongoing research, and was being subjected to verification testing via factorial analysis. This factorialism dovetailed with Zuckerman’s mathematized matrix, translating previously ‘subjective’ and idiosyncratic determinations of character types into tabular data. It also had the advantage of privileging the bodily exterior, of eliding interior states by tying them to objectively measurable physical forms. Furthermore, it was well suited to the kind of triage psychological assessment that the Hull survey represented, enabling a quick and reasonably non-intrusive mapping of potential neurotics without committing the sin of interior probing that might itself be the cause of neurosis (Fraser, ‘Some Illustrative Case Histories’, *c.*1941).
